# Anti-inflammatory effects of Edaravone and Scutellarin in activated microglia in experimentally induced ischemia injury in rats and in BV-2 microglia

**DOI:** 10.1186/s12868-014-0125-3

**Published:** 2014-11-22

**Authors:** Yun Yuan, Hao Zha, Parakalan Rangarajan, Eng-Ang Ling, Chunyun Wu

**Affiliations:** Department of Anatomy and Histology/Embryology, Faculty of Basic Medical Sciences, Kunming Medical University, 1168 West Chunrong Road, Kunming, 650500 People’s Republic of China; Department of Anatomy, Yong Loo Lin School of Medicine, National University of Singapore, Blk MD10, 4 Medical Drive, Singapore, 117597 Singapore

**Keywords:** Activated microglia, Cerebral ischemia, Edaravone, Scutellarin, Anti-inflammation

## Abstract

**Background:**

In response to cerebral ischemia, activated microglia release excessive inflammatory mediators which contribute to neuronal damage. Therefore, inhibition of microglial over-activation could be a therapeutic strategy to alleviate various microglia-mediated neuroinflammation. This study was aimed to elucidate the anti-inflammatory effects of Scutellarin and Edaravone given either singly, or in combination in activated microglia in rats subjected to middle cerebral artery occlusion (MCAO), and in lipopolysaccharide (LPS)-induced BV-2 microglia. Expression of proinflammatory cytokines, including tumor necrosis factor-alpha (TNF-α), interleukin-1 beta (IL-1β), and inducible nitric oxide synthase (iNOS) was assessed by immunofluorescence staining and Western blot. Reactive oxygen species (ROS) and nitric oxide (NO) levels were determined by flow cytometry and fluorescence microscopy, respectively.

**Results:**

*In vivo*, both Edaravone and Scutellarin markedly reduced the infarct cerebral tissue area with the latter drug being more effective with the dosage used; furthermore, when used in combination the reduction was more substantial. Remarkably, a greater diminution in distribution of activated microglia was observed with the combined drug treatment which also attenuated the immunoexpression of TNF-α, IL-1β and iNOS to a greater extent as compared to the drugs given separately. *In vitro,* both drugs suppressed upregulated expression of inflammatory cytokines, iNOS, NO and ROS in LPS-induced BV-2 cells. Furthermore, Edaravone and Scutellarin in combination cumulatively diminished the expression levels of the inflammatory mediators being most pronounced for TNF-α as evidenced by Western blot.

**Conclusion:**

The results suggest that Edaravone and Scutellarin effectively suppressed the inflammatory responses in activated microglia, with Scutellarin being more efficacious within the dosage range used. Moreover, when both drugs were used in combination, the infarct tissue area was reduced more extensively; also, microglia-mediated inflammatory mediators notably TNF-α expression was decreased cumulatively.

**Electronic supplementary material:**

The online version of this article (doi:10.1186/s12868-014-0125-3) contains supplementary material, which is available to authorized users.

## Background

Ischemic stroke constitutes most of all strokes and is caused by obstruction of blood flow to the brain, which would initiate a complex cascade of metabolic alterations, including release of reactive oxygen species and inflammatory cytokines, activation of complement proteins etc. This would then exacerbate neuroinflammation and ultimately cause neuronal death. The innate immune response to induce postischemic inflammation is undoubtedly the hallmark feature for the progression of cerebral ischemia injury [1]. The key cell players in this are the activated microglia which can act as sensors to detect abnormal alterations in response to internal and external insults.

Microglial cells are the resident immune cells that mediate neuroinflammation in the central nervous system (CNS) [2] In neurodegenerative diseases and stroke, they are activated and engaged in different functions such as phagocytizing the toxic cellular debris, producing proinflammatory cytokines and enhancing neuronal survival by release of trophic factors [3]. In chronic activation, microglia are thought to contribute to neuronal damage via release of excessive proinflammatory cytokines and/or cytotoxic factors, such as nitric oxide (NO), tumor necrosis factor-α (TNF-α), interleukin-1β (IL-1β), and reactive oxygen species (ROS) [4,5]. As a corollary, inhibition or suppression of microglia to prevent over-reaction and inflammatory response of microglia may prove to be an efficacious therapeutic strategy to alleviate the progression of the neurological diseases.

In the search for potential drugs that may effectively suppress overt microglial activation, attention has recently been drawn to Edaravone and Scutellarin. Edaravone (3-methyl-1-phenyl-2-pyrazolin-5-one), a free radical scavenger that is currently used in the treatment of acute ischemic stroke as a neuroprotective reagent, has been shown to significantly reduce the infarct size, improve neurological scores, and decrease ROS generation [6]. More specifically, it can counteract toxicity from activated microglia [7]. Neuroinflammation in middle cerebral artery occlusion (MCAO) may be attenuated by Edaravone which acts through suppression of expression of proinflammatory cytokines in activated microglia [8].

**Figure 1:**
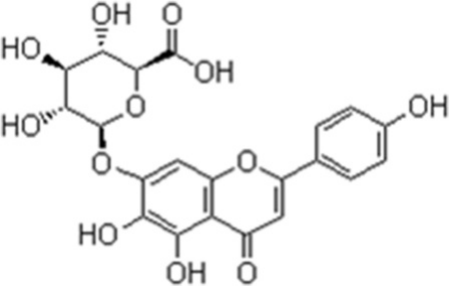
**Chemical structure of Scutellarin.** Image adapted from product supplier’s (Shanghai Winherb Medical Technology) website: http://www.winherb.cn/offer/134772738.html.

Scutellarin (4,5,6-trihydroxyflavone-7-glucuronide) is the major active component (Figure 1) extracted from *Erigeron breviscapus* (Vant.) Hand-Mazz [9]. It is one of the widely used herbal medicines in China for treatment of ischemic cerebrovascular diseases. Studies have shown that Scutellarin has neuroprotective effects because of its antioxidant [10,11] and antiapoptotic [9] properties. Very interestingly, Scutellarin exerts anti-inflammatory action in several animal models [12,13]. In addition, it can inhibit lipopolysaccharide (LPS)-induced production of proinflammatory mediators such as NO, TNF-α, IL-1β and ROS in rat primary microglia or BV-2 mouse microglial cell line [14]. It decreases the number of activated microglia and reduces the expression of Toll-like receptor 4 (TLR4), nuclear factor kappa B (NF-*κ*B) p65 and inflammatory mediators [15].

The above studies have shown that both Edaravone and Scutellarin have anti-inflammatory effects in activated microglia. It remains to be determined which of the two common drugs is more potent in its anti-inflammatory effect in activated microglia, and whether there would be a cumulative therapeutic effect when both drugs were used in combination. This study was therefore aimed to investigate the anti-inflammatory effect of Edaravone and Scutellarin used either singly or in combination in experimentally induced cerebral ischemia, and *in vitro* in the BV-2 microglial cells. We sought to determine if a combination of Edaravone and Scutellarin at appropriate dosage may represent a more efficacious therapeutic strategy for treatment of neurodegenerative diseases in which activated microglia are implicated.

## Results

### Changes in infarct size in MCAO rats given Edaravone and/or Scutellarin treatment

**Figure 2:**
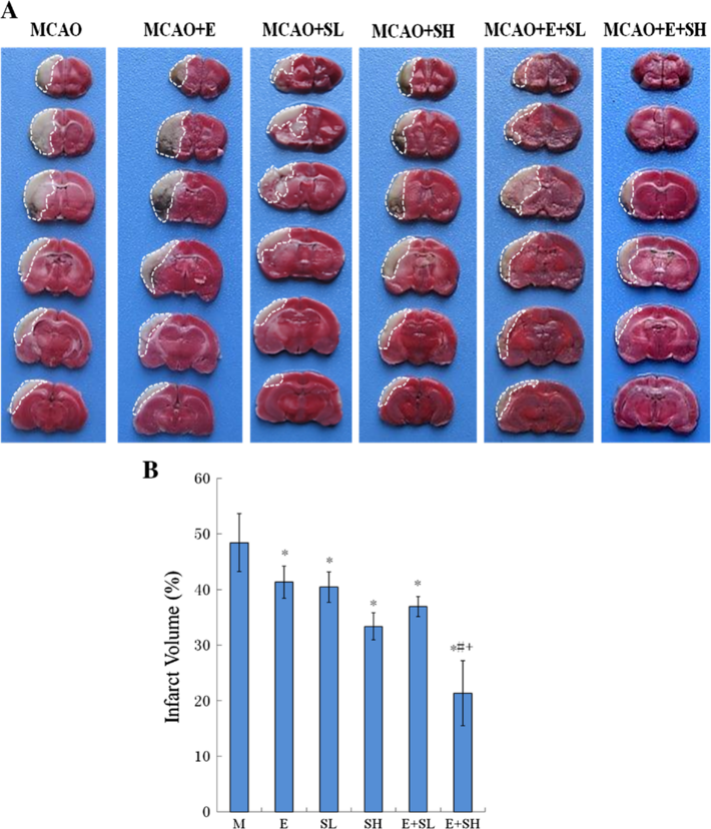
**Reduction in size of infarct zone was observed in the brain cortices of MCAO rats following treatment with drugs Edaravone (E), Scutellarin (S) and E + S. (A)** Triphenyl tetrazolium chloride (TTC) staining showing a marked decrease in the infarct size in brain sections following treatment of MCAO rats with drug E alongside low (SL) and high (SH) dosages of drug S and killed at 3 days after MCAO (n = 5 for each group). (**B)** Significant differences in infarct volume in MCAO (M) rats compared with other group was expressed as *p <0.05. The infarct volume in MCAO rat brains treated with E + SH shows marked differences compared with that treated with E or SH, ^#+^p <0.05. Each bar represents mean ± SD.

A large infarct area was observed in the ipsilateral cerebrum in the MCAO rats at 3 days after MCAO. Compared with this group, the infarct area of the cerebral cortex was markedly reduced by Edaravone (E) or Scutellarin (S) treatment or a combination of both drugs (Figure 2A). Treatment of MCAO rats with Edaravone along with high dose Scutellarin (E + SH) markedly reduced the infarct volume. There were no apparent differences between Edaravone combined with low dose Scutellarin (E + SL) group and Edaravone group (E) or Scutellarin low dose group (SL). On the other hand, the infarct volume in E + SH group was significantly decreased compared with Scutellarin high dose group (SH) (Figure 2B).

### Microglia were activated after MCAO but were reduced in cell numbers following treatment with drugs

**Figure 3:**
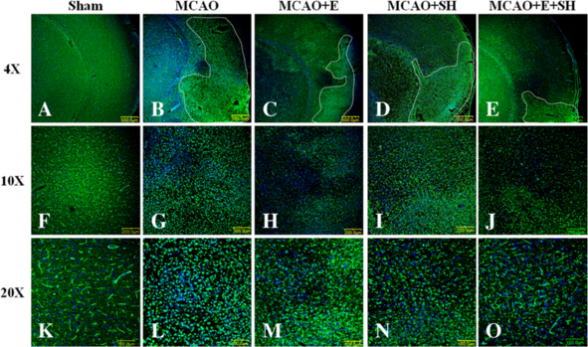
**Reduction in infarct size and distribution of lectin** ^**+**^ **activated microglia in the cerebral cortex of MCAO rat brains following treatment with drugs E, SH and E + SH.** Confocal images showing the distribution of lectin^+^ (green) microglia in the cerebral cortex of MCAO rat brains with drugs E, SH and E + SH compared to sham-operated control (n = 5 for each group). Dotted lines delineate the size of infarct zone in the tissue. Note that the infarct size is considerably reduced in MCAO rat brains treated with drugs E, SH and especially with E + SH **(C-E)** in comparison to MCAO **(B)**. Additionally, the number of lectin^+^ activated microglia is noticeably reduced in MCAO rat brains treated with drugs E, S and E + S as seen in high magnification confocal images **(H-J and M-O)** in comparison to MCAO **(G, L)**. DAPI – blue. Scale bars in **A-E**: 500 μm, **F-J**: 200 μm, **K-O**: 100 μm.

The infarct size was considerably reduced in MCAO rat brains at 3 days treated with drugs E, S and E + S in comparison to untreated MCAO rats. The activated microglia, in large numbers, were observed in the ipsilateral cerebral cortex of MCAO rat brain without drugs treatment. The incidence of activated microglia was noticeably reduced in MCAO rat brains when treated with drug E, S and E + S and this was accompanied by a decrease in the infarct zone being most pronounced in the last mentioned group (Figure 3).

### Edaravone and Scutellarin separately or in combination reduced the expression of inflammatory mediators in MCAO rats

**Figure 4:**
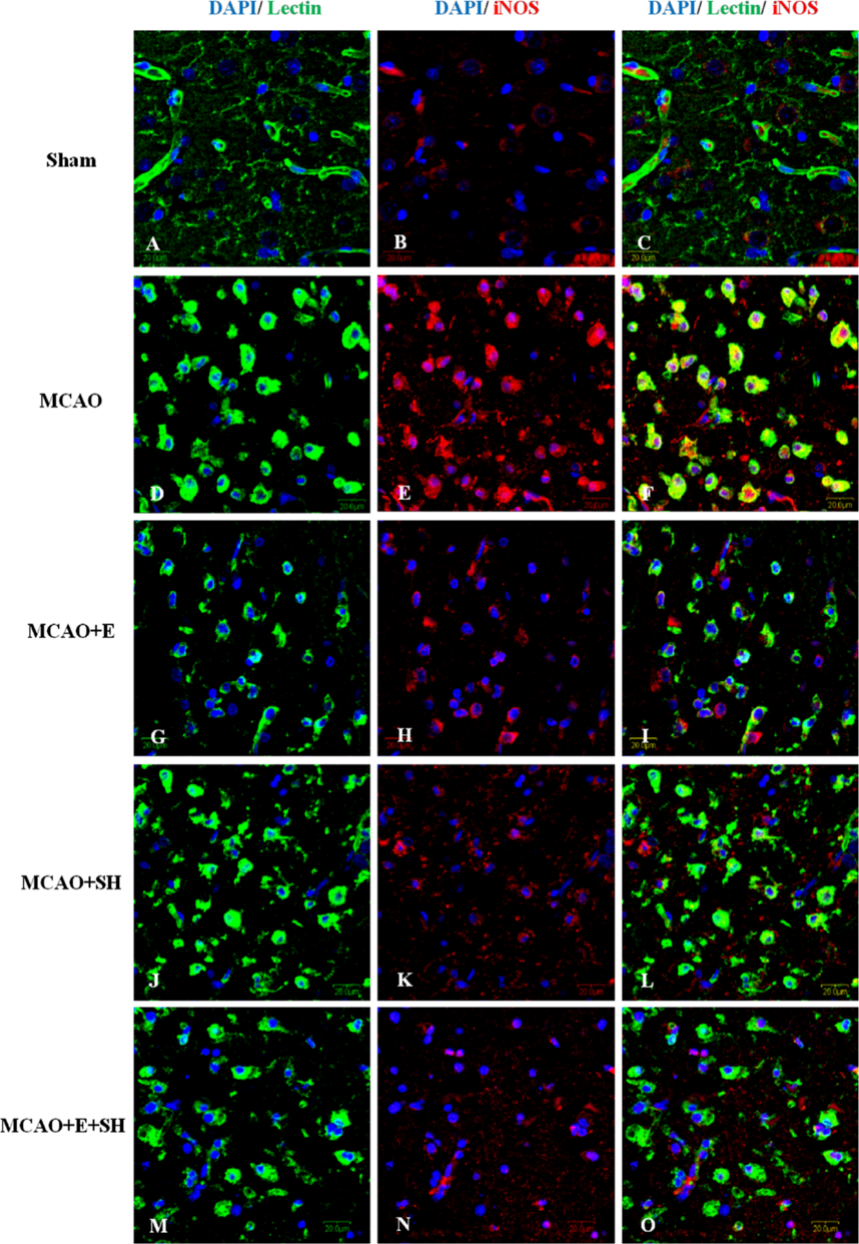
**Treatment of MCAO rats with drugs E, SH and E + SH resulted in the reduction of iNOS expression in activated microglia.** Confocal images showing the expression of iNOS (red) in lectin^+^ microglia (green) in the penumbral zones of MCAO rat brain **(D-F)** and following treatment with E **(G-I)**, SH **(J-L)** and E + SH **(M-O)** (n = 5 for each group). Increase in iNOS expression **(E)** can be observed in the activated microglia **(D)** in MCAO rat brain. A marked reduction of iNOS expression **(H, K)** was observed in activated microglia **(G, J)** 3 days following treatment of MCAO rats with drugs E and SH. Further, iNOS expression **(N)** was hardly detectable in activated microglia **(M)** when MCAO rats were treated with a combination of the two drugs. DAPI – blue. Scale bars in **A-O**: 20 μm.

**Figure 5:**
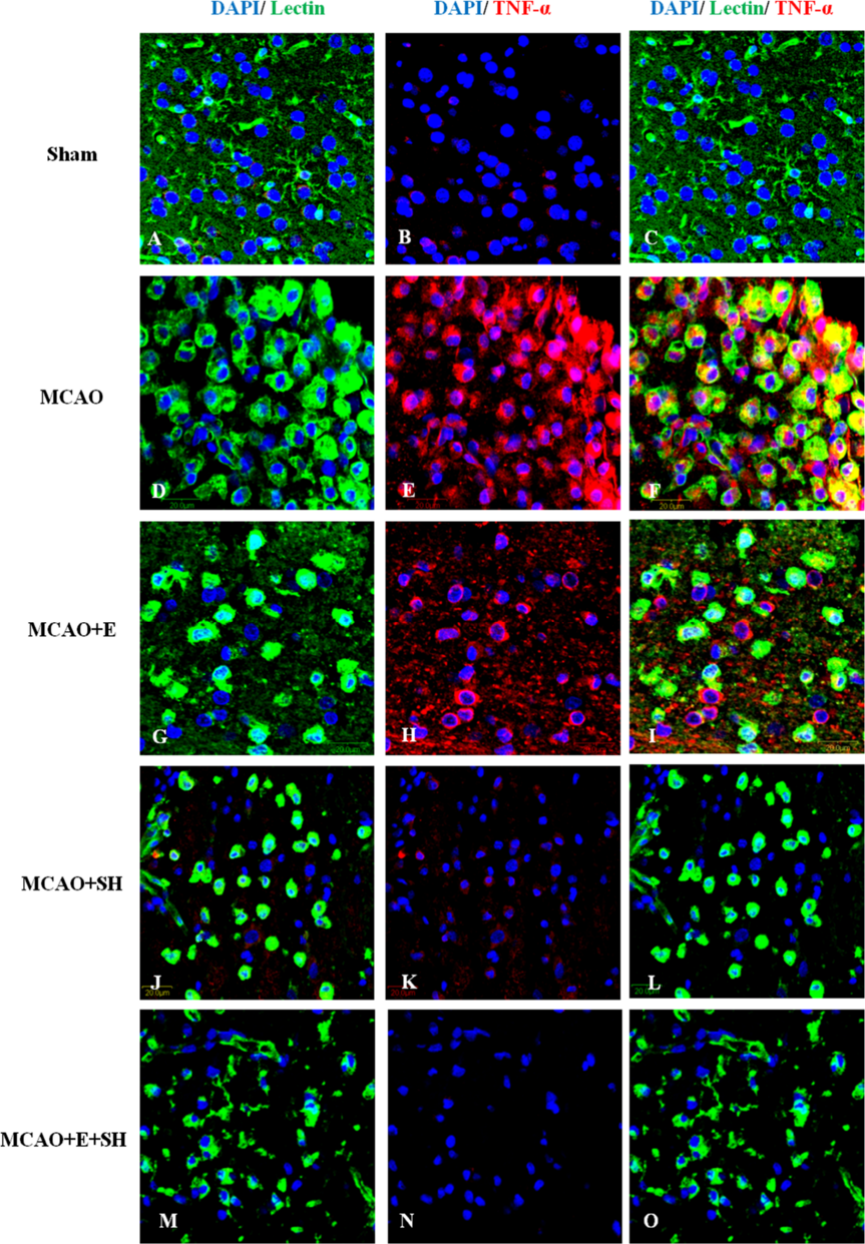
**Treatment of MCAO rats with drugs E, SH and E + SH resulted in the reduction of TNF-α expression in activated microglia.** Confocal images showing the expression of TNF-α (red) in lectin^+^ microglia (green) in the penumbral zones of MCAO rat brain **(D-F)** and following treatment with E **(G-I)**, SH **(J-L)** and E + SH **(M-O)** (n = 5 for each group). A drastic increase in the expression of TNF-α **(E)** can be observed in the activated microglia **(D)** in MCAO rat brain. A marked reduction of TNF-α expression **(H, K)** was observed in activated microglia **(G, J)** 3 days following treatment of MCAO rats with drugs E and SH. Further, TNF-α expression **(N)** was negligible in activated microglia **(M)** when MCAO rats were treated with a combination of the two drugs. DAPI – blue. Scale bars in **A-O**: 20 μm.

**Figure 6:**
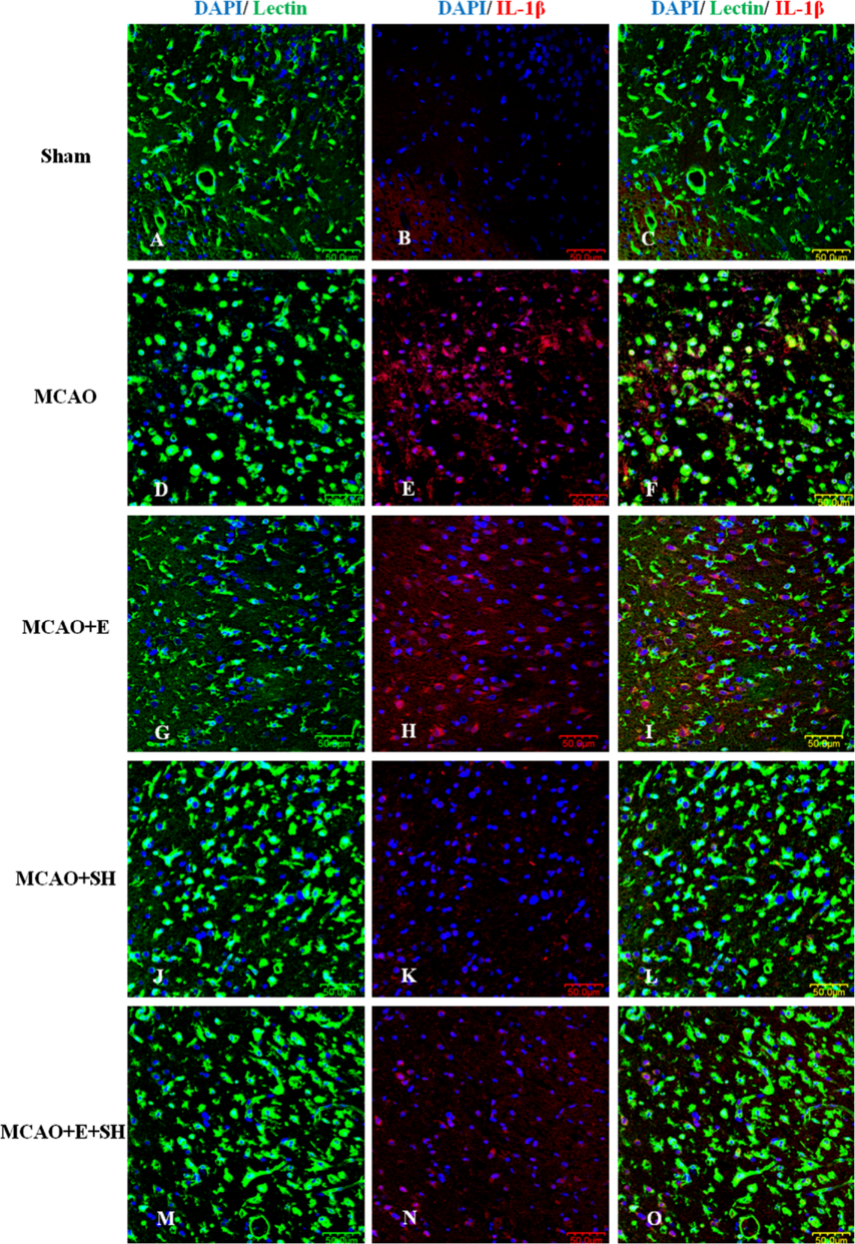
**Treatment of MCAO rats with drugs E, SH and E + SH resulted in the reduction of IL-1β expression in activated microglia.** Confocal images showing the expression of IL-1β (red) in lectin^+^ microglia (green) in the penumbral zones of MCAO rat brain **(D-F)** and following treatment with E **(G-I)**, SH **(J-L)** and E + SH **(M-O)** (n = 5 for each group). A noticeable increase in IL-1β expression **(E)** can be observed in the activated microglia **(D)** in MCAO rat brain. IL-1β expression **(H, K)**, however, was depressed in activated microglia **(G, J)** 3 days following treatment of MCAO rats with drugs E and SH. Also, IL-1β expression **(N)** was almost totally abolished in activated microglia **(M)** when MCAO rats were treated with a combination of the two drugs. DAPI – blue. Scale bars in **A-O**: 50 μm

To investigate the anti-inflammatory effects of Edaravone and Scutellarin on activated microglia, we examined the production of inflammatory cytokines (TNF-α and IL-1β) and iNOS in MCAO rats given the treatment of both drugs either separately or in combination by double immunofluorescence staining in MCAO rats given either low or high dose of Scutellarin. Here we show the images of Scutellarin high dose (SH) only. iNOS immunofluorescence in activated microglia in the penumbral zones was noticeably enhanced after MCAO, but it was markedly reduced at 3 days following treatment with E, SH and E + SH. This was conspicuous in MCAO + SH and especially so in MCAO + E + SH rats in which iNOS expression in activated microglia was virtually abolished (Figure 4). The expression of TNF-α (Figure 5) and IL-1β (Figure 6) paralleled with that of iNOS after treatment with E, SH or E + SH. Thus, in MCAO rats given SH or E + SH treatment notably in the latter and killed at 3 days post-operation, TNF-α and IL-1β immunoexpression in lectin labeled activated microglia was obliterated. In MCAO rats killed at 7 days, iNOS and TNF-α immunofluorescence that was augmented following ischemia was attenuated with E, SH or E + SH treatment (see Additional file 1: Figure S1, Additional file 2: Figure S2). An additional feature was the profuse ramification of microglia at this time point. In general, immunofluorescence of iNOS and TNF-α was less intense compared with that at 3 days.

**Figure 7:**
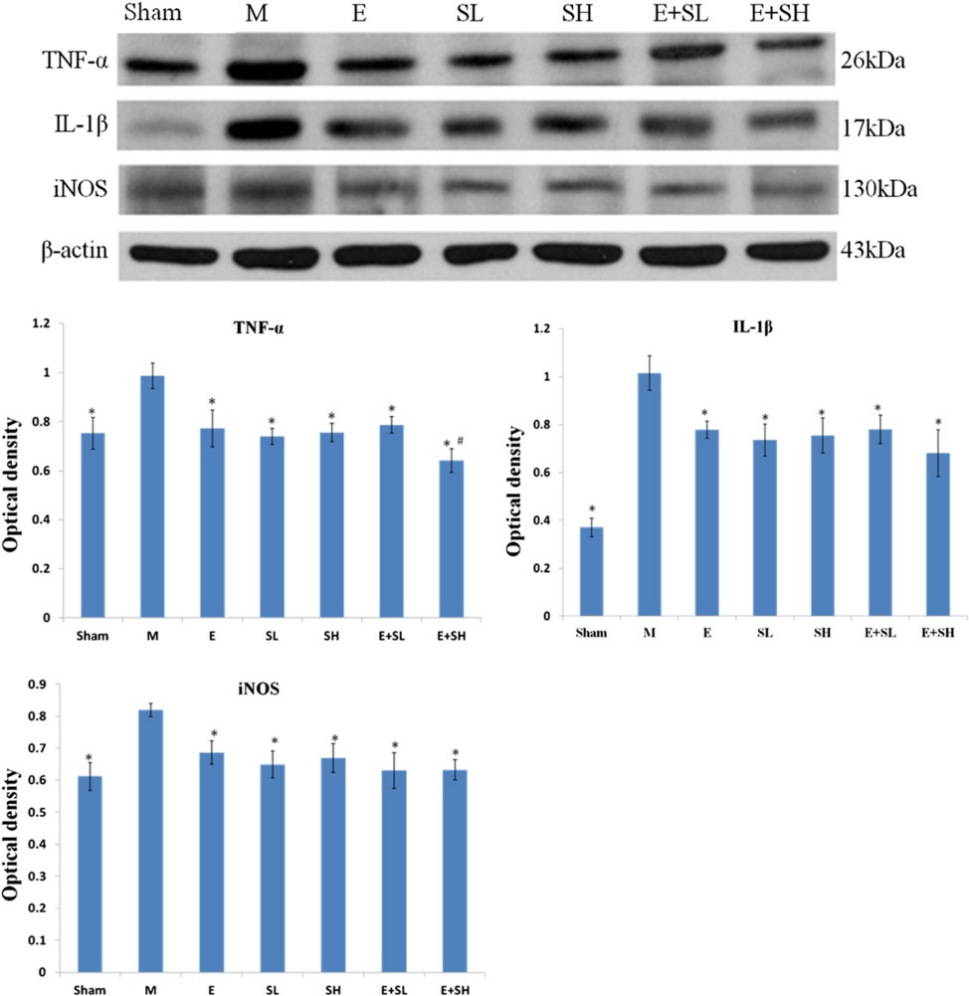
**Protein expression of inflammatory cytokines and iNOS was decreased in MCAO rat brains following treatment with E, S and E + S.** The expression levels of TNF-α, IL-1β and iNOS in MCAO rat brains are depressed significantly at 3 days following treatment with E, S and E + S when compared with the MCAO ( n = 5 for each group). Significant differences in protein levels between MCAO and drugs used rats are expressed as *p <0.01. TNF-α protein expression level of the E + SH group was most drastically suppressed following MCAO when compared with the other groups. Significant difference is expressed as ^#^p <0.05. The values represent the mean ± SD in triplicate.

Western blot analysis also showed that the protein expression of iNOS, TNF-α and IL-1β was obviously suppressed at 3 days after treatment with drugs (Figure 7). It is striking that TNF-α expression in MCAO rats given combined E + SH treatment showed the most drastic reduction when compared with drugs used separately (p <0.05). The expression level of TNF-α in MCAO rats treated with E + SH was reduced by about 35%, compared with 24% of treatment with SH alone, i.e. a further decrease by 11%. Likewise, the expression level of IL- 1β and iNOS in combined drug treatment (E + SH) was further suppressed by 7% and 4%, respectively, when compared with rats treated with SH alone.

### Cell viability assay of BV-2 cells

We chose to perform our *in vitro* studies on BV-2 cell line instead of primary cultured microglial cells to ensure that we obtained enough cells for analyzing cell viability following drug treatment and also obtained adequate amounts of protein for Western blot analyses. The cytotoxicity data was obtained by the MTS assay for the effect of Edaravone and Scutellarin on BV-2 cells. A combined concentration of Edaravone (in the range of 50 μM to 200 μM) and Scutellarin (in the range of 0.27 mM to 1.1 mM) did not result in any significant cell death (Additional file 3: Figure S3). In this study we have used Edaravone at 100 μM with Scutellarin at 0.54 mM for all subsequent analysis.

### Edaravone and Scutellarin separate treatment or in combination reduced the expression of inflammatory cytokines and iNOS in LPS-induced BV-2 microglia

**Figure 8:**
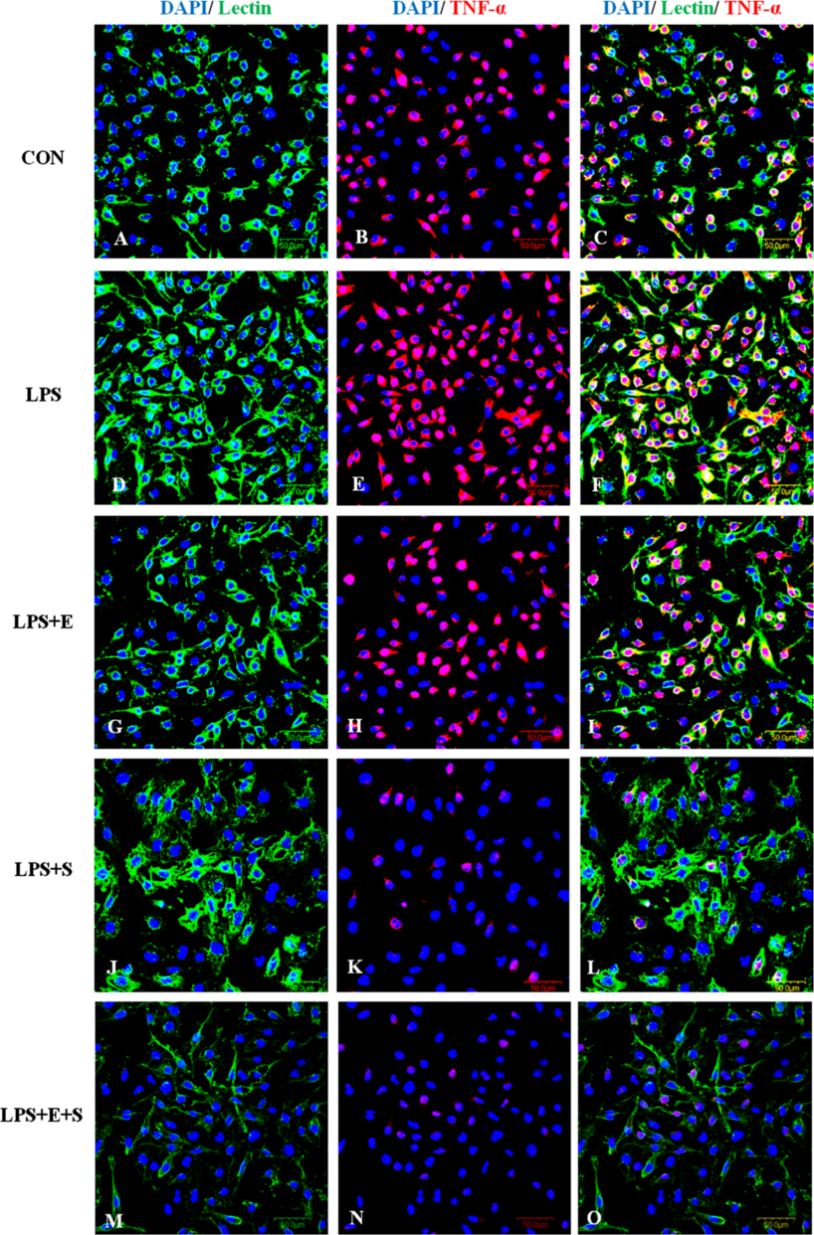
**TNF-α expression was suppressed following treatment of LPS-activated microglia with drugs E, S and E + S** ***in vitro*** **.** Confocal images show an upregulation of TNFα **(E)** in LPS-activated BV-2 microglia **(D)** in comparison to control **(A-C)**. Addition of drugs E and S led to decrease in the expression of TNF-α **(H, K)** in LPS-activated BV-2 microglia **(G, J)**. TNF-α was hardly detected in activated microglia when treated with a combination of drugs E and S **(N)**. Following treatment with drugs E and S, activated microglia emitted thin cytoplasmic processes **(M)** reminiscent of the ramified microglia *in vivo*. DAPI – blue. Scale bars in **A-O**: 50 μm

**Figure 9:**
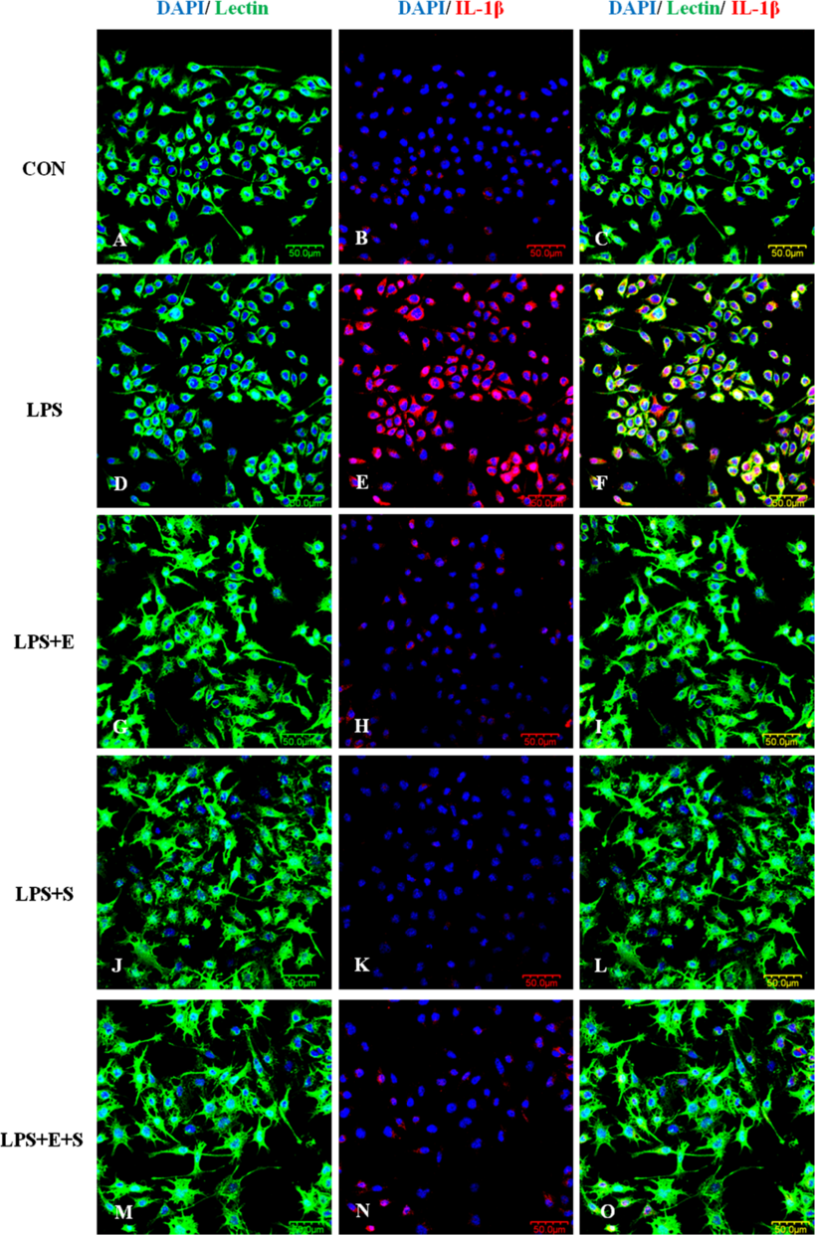
**IL-1β expression was suppressed following treatment of LPS-activated microglia with drugs E, S and E + S** ***in vitro*** **.** Confocal images show an upregulation of IL-1β **(E)** in LPS-activated BV-2 microglia **(D)** in comparison to control **(A-C)**. IL-1β was hardly detected **(H, K, N)** in activated microglia treated with drugs E, S and especially so with E + S **(G, J, M)**. DAPI – blue. Scale bars in **A-O**: 50 μm.

**Figure 10:**
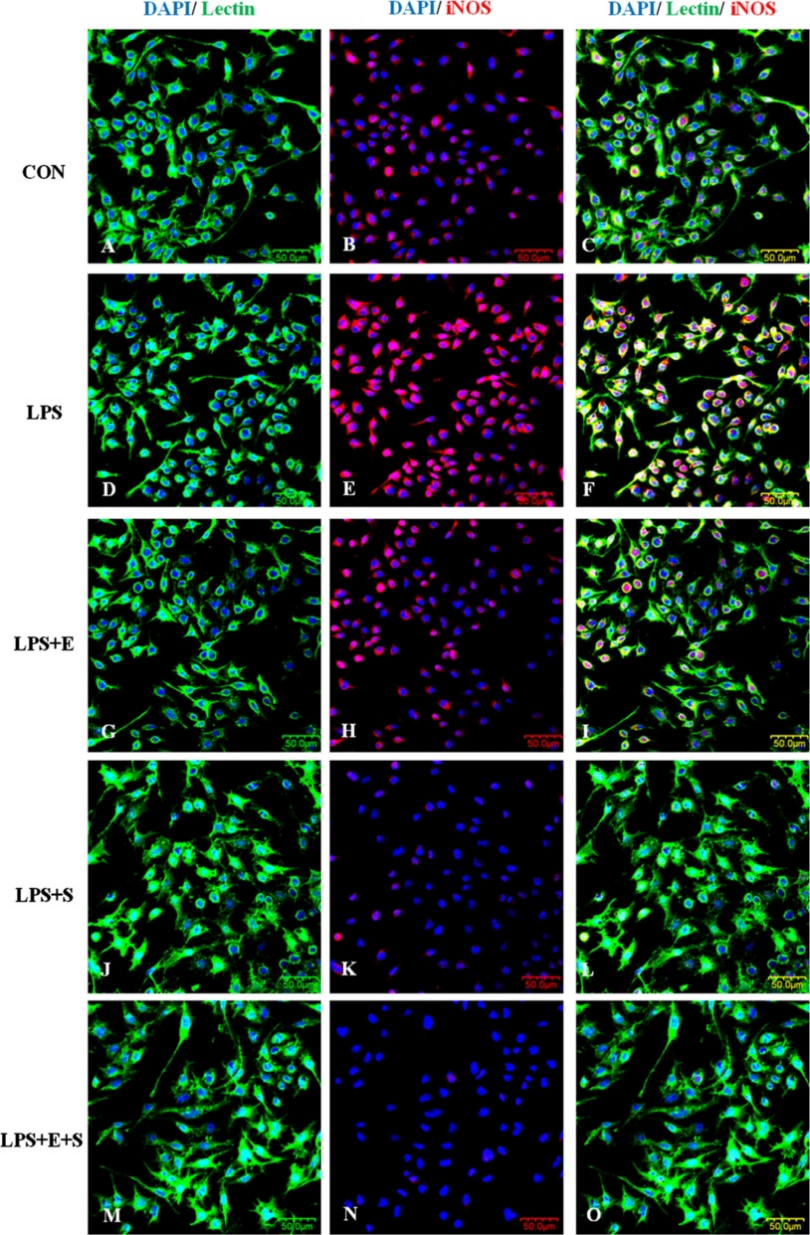
**iNOS expression was drastically suppressed following treatment of LPS-activated microglia with drugs E, S and E + S** ***in vitro*** **.** Confocal images show an upregulation of iNOS **(E)** in LPS-activated BV-2 microglia **(D)** in comparison to control **(A-C)**. Addition of drug E resulted in decrease in iNOS expression **(H)** in LPS-activated BV-2 microglia **(G)**. iNOS expression was obliterated in activated microglia treated with drug S **(K)** notably with a combination of drugs E and S **(N)**. DAPI – blue. Scale bars in **A-O**: 50 μm.

Consistent with results *in vivo*, changes in the inflammatory mediators, including TNF-α, IL-1β and iNOS, whose immunoexpression was also observed in LPS-induced BV-2 cells. TNF-α, IL-1β and iNOS immunofluorescence intensity was markedly augmented versus controls when the cells were subjected to LPS, but was suppressed in LPS-activated microglia pretreated with E, S and E + S (Figures 8, 9, 10). The expression of these inflammatory mediators was clearly diminished in activated BV-2 cells that were pretreated with S or a combination of the both drugs.

**Figure 11:**
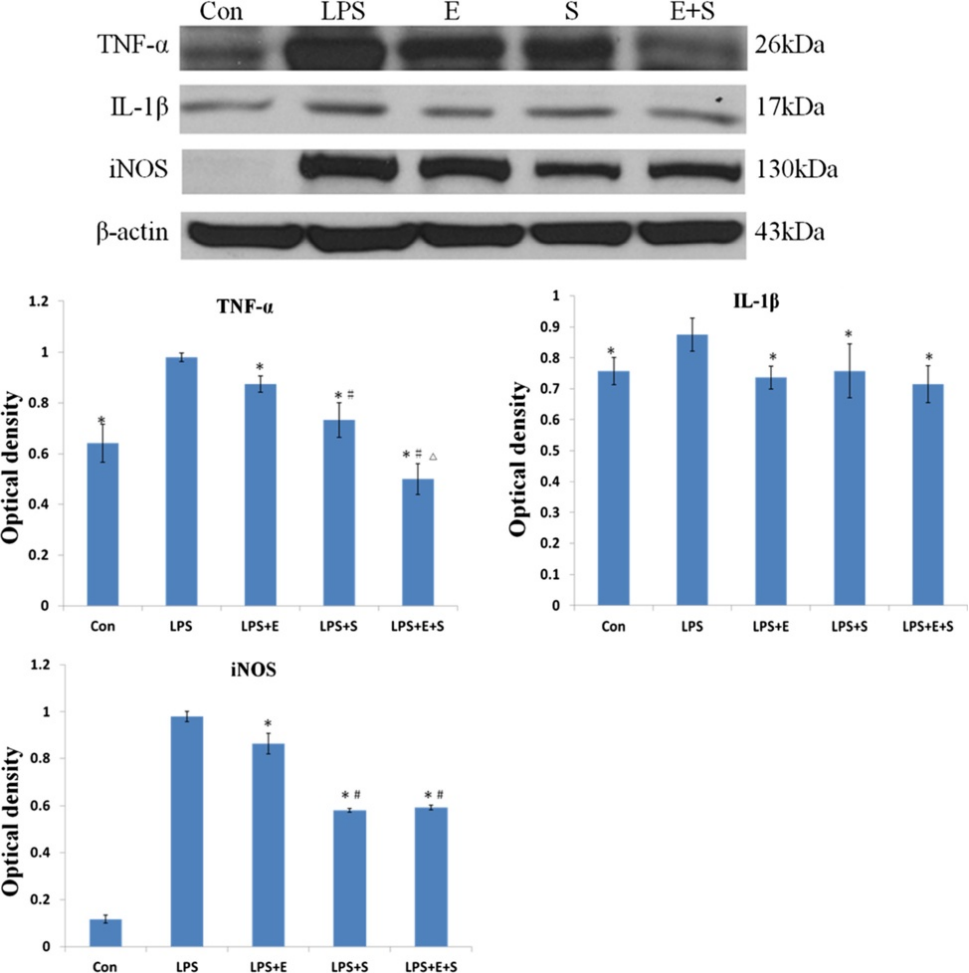
**Protein expression of inflammatory cytokines and iNOS was decreased in LPS-activated microglial cells following treatment with E, S and E + S.** The expression levels of TNF-α, IL-1β and iNOS in LPS-activated microglial cells are depressed significantly following treatment with E, S and E + S. Significant differences in protein levels between LPS and drugs used groups are expressed as *p < 0.05. TNF-α and iNOS protein expression level in LPS + S group and LPS + E + S group was markedly suppressed when compared with the LPS + E group. Furthermore, the expression of TNF-α in LPS + E + S BV-2 cells was significantly lower than other groups. Significant differences in protein levels are expressed as ^#^p < 0.05 and ^△^p <0.01. The values represent the mean ± SD in triplicate.

The protein expression of iNOS, TNF-α and IL-1β was decreased in BV-2 cells pretreated with drugs. In this connection, the expression of TNF-α was further depressed when E + S were used in combination when compared with the other groups, a phenomenon that is consistent with the result *in vivo* (Figure 11). The expression level of TNF-α in LPS-induced BV-2 cells with E + S was reduced by about 49%, compared with 25% reduction in cells given treatment with S alone. This amounted to a further decrease by about 24%. The expression level of IL- 1β and iNOS in combined drug treatment was further suppressed by 3% and 27%, respectively, when compared with E treatment alone. The expression level of IL- 1β in combined drug treatment was further suppressed by 5% when compared with S treatment alone, but iNOS expression in drug combination group was not significantly lower than that of S used alone.

### Edaravone and Scutellarin separate treatment or in combination reduced the expression of ROS and NO in LPS-induced BV-2 cells

**Figure 12:**
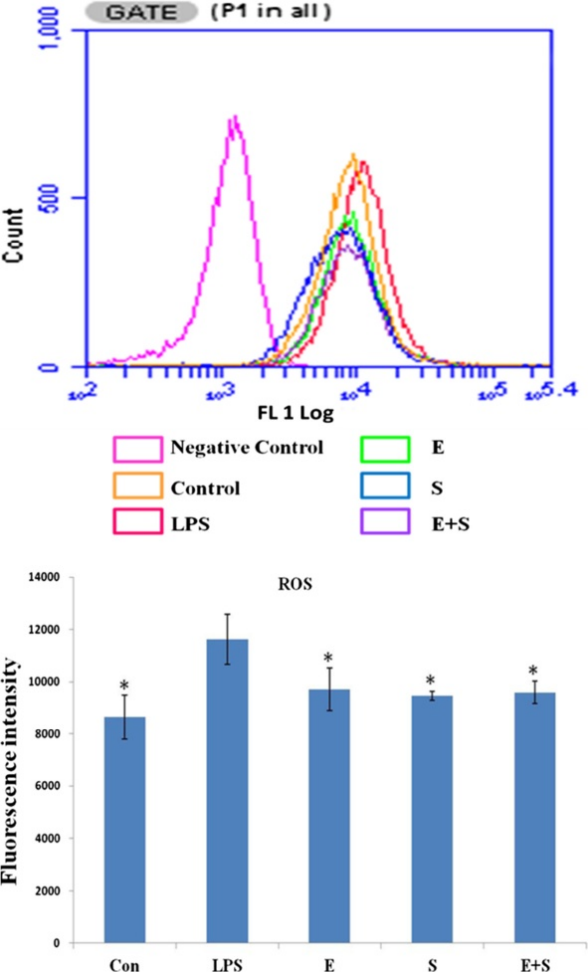
**ROS expression was reduced in LPS-activated microglial cells following treatment with E, S and E + S.** Intracellular ROS in LPS-activated BV-2 microglial cells following treatment with E, S and E + S was measured. The upper panel shows cell counts (y-axis) and log10 expression of fluorescence intensity (x-axis). The lower panel is a bar graph showing a significant change in the fluorescence intensity of intracellular ROS production following the above treatments when compared with the LPS. Note the ROS production, which is increased after LPS stimulation, is significantly decreased after pretreatment with E, S and E + S. Significant differences in protein levels are expressed as ^*^p <0.05.

**Figure 13:**
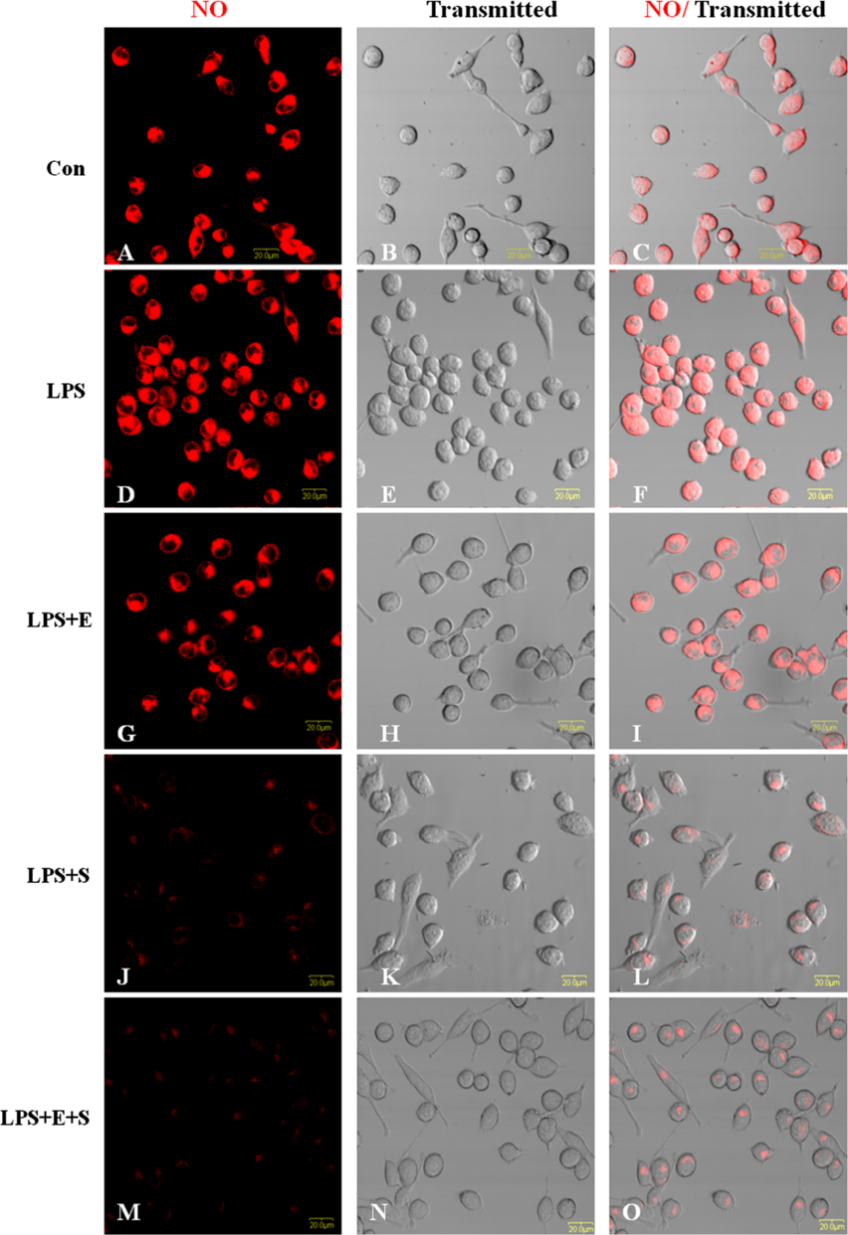
**NO expression was reduced in LPS-activated microglial cells following treatment with E, S and E + S.** Intracellular NO in LPS-activated BV-2 microglial cells following treatment with E, S and E + S was measured. Confocal images showing the expression of NO (red) in LPS-induced microglia (transmitted) **(D-F)** and following treatment with E **(G-I)**, S **(J-L)** and E + S **(M-O)**. Increase in NO expression **(D)** can be observed in LPS-activated BV-2 microglia **(E)** in comparison to control **(A-C)**. Addition of drug E led to decrease in the expression of NO **(G)** in LPS-activated BV-2 microglia **(H)**. NO expression was significantly suppressed in activated microglia treated with drug S **(J)** and a combination of drugs E and S **(M)**. Scale bars in **A-O**: 20 μm.

Intracellular ROS and NO in LPS-activated BV-2 microglia following treatment with E, S and E + S was measured. ROS production was increased in LPS-induced BV-2 microglia, and significantly decreased in cells pretreated with E, S and E + S (Figure 12). NO production was markedly increased in LPS-induced BV-2 cells, and was observably reduced when the cells were pretreated with E, S and in combination. The expression of NO in S or E + S treatment group was clearly reduced especially in the latter when compared with E treatment group (Figure 13).

## Discussion

Neuroinflammation is a key contributor in the ischemic cascade after cerebral ischemia that leads to neuron damage and death. Activated microglia in inflammatory response have both beneficial and detrimental functions in the nervous system. During neuroinflammation, activated microglia remove cellular debris or invading pathogens, and release neurotrophic factors that regulate the microenvironment [16]. On the other hand, activated microglial cells are also elicited to produce a plethora of proinflammatory mediators, including TNF-α, IL-1β, ROS etc., which have been implicated in the pathogenesis of different neurodegenerative diseases, including Alzheimer’s disease [17], Huntington’s disease [18], Parkinson’s disease [19], stroke [20] and hypoxic insults [21]. Therefore, a prevalent view is that activated microglia can aggravate the injury and subsequent neurodegeneration and they may serve as a prime therapeutic target in a wide variety of CNS diseases.

Here we examined the anti-inflammatory action of Edaravone and Scutellarin in activated microglia. Both Edaravone and Scutellarin reduced the infarct size in brain cortices of MCAO rats, inhibited production of pro-inflammatory mediators (TNF-α, IL-1β, NO and ROS) in MCAO rats and LPS-induced BV-2 microglial cells, as well as suppressed the experimentally induced increased expression of iNOS. Activated microglia appeared to emit many thin cytoplasmic processes when treated with Edaravone and Scutellarin. A possible explanation for this would be that both drugs had promoted the ramification of microglia whose ramified phenotype might represent a less active state. On the other hand, the possibility that Edaravone and Scutellarin can suppress the activated microglia by causing the cells to become more resistant to transformation into an amoeboid form, presumably a more active state, is considered. In any event, a significant diminution in distribution of activated microglia was evident in rats treated with both drugs combined. Furthermore, we have also provided the first morphological evidence that in comparison to Edaravone, Scutellarin was more efficacious in suppressing the expression levels of inflammatory mediators in activated microglia with the dosage used. Additionally, the two drugs in combination cumulatively depressed the expression of TNF-α both *in vivo* and *in vitro*. The results suggest that Scutellarin is more potent in therapeutic potential for various microglia mediated neuroinflammatory diseases.

Edaravone is a synthetic small-molecule free-radical scavenger. It has been reported to be effective in inhibiting the inflammatory responses [22], brain edema [23], ROS generation, and oxidative tissue damage [24]. Edaravone currently is being used clinically to treat stroke patients. Recently, Edaravone has been shown to possess neuroprotective and antioxidative effects on the brain after traumatic brain injury both in rat models and in patients [25-27]. The likely underlying mechanism for this is via inhibiting oxidative stress, leading to a decreased inflammatory response, and thereby reducing neuronal death and improving neurological function. Many *in vivo* experiments have similarly reported that Edaravone could mitigate microglial activation and suppress the production of proinflammtory meditors by activated microglia [8,28]. Here, we confirmed the efficacy of Edaravone in inhibiting the expression levels of various inflammatory mediators in activated microglia as shown by Western blot analysis and immunofluorescence labeling.

It has been reported that Scutellarin could improve neuronal injury and had protective effect in rat cerebral ischemia at least related to its antioxidant property [29]. The neuroprotective effect of Scutellarin was associated with inhibition of the apoptosis-inducing factor pathway [9]. Recently, some studies have extended that Scutellarin could inhibit production of proinflammatory mediators induced by LPS in rat primary microglia or BV-2 microglial cells [14]. The present results are consistent with this. It is unequivocal from the present results that Scutellarin with the dosage used based on cell viability assay showed a greater potency in comparison to Edaravone in its anti-inflammation in activated microglia; hence, it is suggested that Scutellarin is endowed with a better therapeutic potential for various microglia mediated neuroinflammatory diseases.

While both Edaravone and Scutellarin have shown great efficacy in their anti-inflammatory effect in activated microglia, the potency of both drugs either administered separately or in combination has not been explored. We show here that when compared with Edaravone, Scutellarin was more effective in decreasing the infarct volume in MCAO rats. Very strikingly, Scutellarin alone suppressed the expression levels of inflammatory mediators to a greater extent when compared to Edaravone in activated microglia. Interestingly, the two drugs in combination cumulatively depressed the expression of inflammatory mediators notably TNF-α in activated microglia. Moreover, Edaravone when combined with a high dose of Scutellarin decreased the infarct volume extensively in MCAO rats. On the other hand, expression of other markers was depressed to a lesser extent in activated microglia between Scutellarin used separately or when combined with Edaravone. The possible explanation for this would be that expression of inflammatory mediators and changes in cerebral infarct volume are not synchronized and there are other mechanisms involved in response to brain injury. Notwithstanding, Scutellarin and Edaravone when used in combination can produce a cumulative protective effect in ischemia brain injury.

In the present results, the infarct volume of the cerebral cortex in MCAO rats was obviously decreased when treated with Edaravone and Scutellarin separately or in combination. Associated with this was the drastic diminution in numbers of activated microglia. Recent studies suggest that Toll-like receptors (TLRs), especially TLR2, may have a key role in the progression of brain damage induced by cerebral ischemia [30-33]. It has been documented that a marked long-term induction of TLR2 expression in microglia activation after transient MCAO, suggesting an important role of TLR2 activation after stroke [31,33]. A significant decrease of the infarct volume in TLR2 deficient mice compared to wild type mice and altered microglia activation profiles has been observed [33,34]. The present results showed that the infarct size was considerably reduced in MCAO rat brain treated with drugs in comparison to untreated MCAO rats. Meanwhile, the activated microglia were not only reduced in numbers but many of them had also appeared ramified phenotype in the ipsilateral cerebral cortex of MCAO rat brain notably in rats given the combined drugs. The possibility is that both Edaravone and Scutellarin can cumulatively suppress the upregulation of TLR2.

We show here that Edaravone and Scutellarin when applied in combination cumulatively decreased the expression of inflammatory mediators, being most pronounced for TNF-α in activated microglia. TNF-α contains soluble and membrane-bind TNF-α [35]. Activated microglial cells are the major producers of soluble TNF-α within the first 6 hours after cerebral ischemia [36-38]. TNF-α converting enzyme (TACE), also known as a disintegrin and metalloprotease 17 (ADAM17) [39], is involved in multiple cell signaling pathways, including p44 mitogen-activated protein kinase (MAPK)-dependent manner [40]. In light of the above, it needs to be further explored whether pretreatment with Edaravone and Scutellarin would directly suppress the expression and function of ADAM17, or act on p38/p44 MAPK to synergisticly decrease TNF-α expression. Scutellarin and Edaravone might target on different sites of TNF-α expression and this should be considered. Studies have shown that inhibition of TNF-α using its antibody reduces infarct volume after MCAO [41,42]. This may offer an explanation on why Edaravone and Scutellarin in combination can markedly reduce the infarct volume after MCAO. Apart from TNF-α, it is interesting to note that there was lesser reduction in the expression of other inflammatory mediators following Edaravone, Scutellarin or combined treatment. One possible explanation for this may be that the expression of these inflammatory mediators has reached the basal levels with either Edaravone or Scutellarin treatment. Therefore, further treatment with two drugs in combination could not suppress the expression further except for TNF-α, which may be more sensitive to the drugs.

A similar phenomenon and possible underlying mechanism are also found on NO expression in BV-2 cells treated. The NO expression was markedly reduced in BV-2 cells treated with Scutellarin or Scutellarin + Edaravone as compared with Edaravone alone within the dosage range as determined by cell viability assay. This is consistent with iNOS expression as manifested by inmunofluorescence labeling and Western blot.

The present morphological evidence and protein analysis indicate that Scutellarin is more potent in suppressing neuroinflammation induced by activated microglia with the dosage used, but the underlying molecular mechanism remains uncertain. Studies have shown that Scutellarin is capable of attenuating the expression of not only those proinflammatory molecules whose expression depends on the activation of NF-κB (a major mediator of microglial inflammatory response), but also those via transcription factor signal transducer and activator of transcription 1α (STAT1α) transcription factor [14]. Recent studies by us have shown that the Notch signaling pathway is involved in microglial activation and microglia-mediated cytokine production by promoting the expression of NF-κB [43,44]. Notch-1 signaling was also identified to regulate microglial activation via NF-κB pathway after hypoxic exposure [45]. Indeed, there is evidence that Notch/NF-κB signaling pathways are involved in inhibition of microglial activation by Scutellarin and production of inflammatory mediators (unpublished data).

## Conclusion

We show here that both Edaravone and Scutellarin could decrease infarct volume, frequency and distribution of activated microglia as well as suppress the production of inflammatory mediators in activated microglia in MCAO rats. Scutellarin appeared to be more potent in its anti-inflammatory effects when compared with Edaravone with the dosage used. Remarkably, the two drugs in combination cumulatively decreased the infarct size in the cerebrum and diminished the ischemia-induced inflammatory mediators being most pronounced for TNF-α expression. Thus, Scutellarin or along with Edaravone may prove to a more efficacious therapeutic strategy for treatment of microglia mediated neurodegenerative diseases, such as stroke.

## Methods

### Ethics statement

This research work has been carried out within an appropriate ethical framework. While handling and use of rats, ethical guidelines as stated in the National Institutes of Health Guide for the Care and Use of Laboratory Animals were adopted. All experimental protocols and use of animals were approved by Kunming Medical University and all efforts were made to minimize the number of rats used and their suffering.

### Animals and experimental groups

A total of 245 adult male Sprague–Dawley (SD) rats weighing 250–280 g were obtained from the Experimental Animal Center of Kunming Medical University. They were randomly divided into sham-operated + saline group (sham), MCAO + saline group (MCAO), MCAO + Edaravone group (5 mg/kg) (E), MCAO + Scutellarin low dose group (50 mg/kg) (SL), MCAO + Scutellarin high dose group (100 mg/kg) (SH), MCAO + Edaravone + Scutellarin low dose group (5 mg/kg Edaravone +50 mg/kg Scutellarin)(E + SL), MCAO + Edaravone + Scutellarin high dose group (5 mg/kg Edaravone +100 mg/kg Scutellarin)(E + SH) (Table 1).

**Table 1:** **Surgical procedures and number of rats used in various treatments**

	**Sham-operated + saline group (sham)**	**MCAO + saline group (MCAO)**	**MCAO + Edaravone group (E)**	**MCAO + Scutellarin low dose group (SL)**	**MCAO + Scutellarin high dose group (SH)**	**MCAO + Edaravone + Scutellarin low dose group (E + SL)**	**MCAO + Edaravone + Scutellarin high dose group (E + SH)**
**TTC**	**n = 5**	**n = 5**	**n = 5**	**n = 5**	**n = 5**	**n = 5**	**n = 5**
**Double inmunofluorescence**	**n = 15**	**n = 15**	**n = 15**	**n = 15**	**n = 15**	**n = 15**	**n = 15**
**Western blot**	**n = 15**	**n = 15**	**n = 15**	**n = 15**	**n = 15**	**n = 15**	**n = 15**
**Total**	**n = 35**	**n = 35**	**n = 35**	**n = 35**	**n = 35**	**n = 35**	**n = 35**

Anesthesia of the rats was achieved by an intraperitoneal injection of sodium pentobarbital (50 mg/kg). The surgical procedure followed that described previously by us [46]. Briefly, a circular aperture 3 mm in diameter was burred in the right parietal bone with a dental drill, and the main trunk of the middle cerebral artery (MCA) was exposed and cauterized. In the sham-operated rats, the same surgical procedure was followed but the MCA was not cauterized.

### Injection of Edaravone and Scutellarin

The rats in the respective groups were given an intraperitoneal injection of Edaravone (5 mg/kg dissolved in saline; Cat. No. H20070051, Jilin, China) and/or Scutellarin (50 mg/kg or 100 mg/kg dissolved in saline; Cat. No.131021, Shanghai Winherb Medical Technology, Shanghai, China) at 2 h before and at 12, 24, 36, 48, 60 h after MCAO; rats were sacrificed at 1, 3 and 7 d after MCAO.

### TTC assessment of infarct size

Thirty five rats were used for assessing infarct size (n = 5 for each group). The rats were killed at 3 d after MCAO. The brains were rapidly removed, frozen at −20°C for 30 min. A total of six 2 mm thick coronal sections of the brain were then cut in a rat brain matrix starting at the frontal pole. This series of brain sections totaling 12 mm in thickness included the entire infarct area caused by the MCAO. The sections were incubated for 30 min and stained with 1% triphenyltetrazolium chloride (TTC) at 37°C protected from light. After this, sections were fixed in 2% buffered formaldehyde solution for 4 h. The cerebral infarct area as outlined in white in MCAO rats as well as in rats after various drug treatments, is depicted in Figure 2. Infarct areas in each section were measured using Image J software. A correction for edema was made according to the following formula: infarct area × (area contralateral hemisphere/area ipsilateral hemisphere) [47,48]. Cerebral infarct volume was measured as a percentage of the total contralateral hemisphere, as calculated with the following formula: total infarct volume = sum of infarct volume of all sections measured (corrected infarct area × 2 mm for each section)/total contralateral hemispheric volume × 100.

### Cell viability assay of BV-2 cells

Cell viability was assessed by CellTiter 96® AQueous One Solution Cell Proliferation Assay kit (Promega, Fitchburg, WI, USA; Cat. No. G3580). To determine the cytotoxic effect of Edaravone and Scutellarin on BV-2 cells, cells were plated into 96-well microplates (10^4^ cells/well) and cultured for 24 h. They were subjected to a combined treatment of Edaravone (in the range of 50 μM to 200 μM) and Scutellarin (in the range of 0.27 mM to 1.1 mM) in each well containing 100 μl of culture medium for 1 h in triplicates. Briefly, 20 μl of MTS(3-(4,5-dimethylthiazol-2-yl)-5-(3-carboxymethoxyphenyl)-2-(4-sulfophenyl)-2H-tetrazolium, inner salt) reagent was added to each well (final concentration, 0.5 mg ml^−1^) and the plate was incubated for an additional 4 h. The optical density (OD) was then read at 490 nm using a microplate reader (GENIOS, Tecan, Switzerland). The assays were performed in triplicate.

### BV-2 cell culture and treatment

BV-2 murine cells were cultured in Dulbecco’s modified Eagle’s medium (DMEM), supplemented with 10% fetal calf serum (FCS) at 37°C in a humidified incubator under 5% CO_2_. The cells were divided into control, LPS-induced, LPS + Edaravone, LPS + Scutellarin, and LPS + Edaravone + Scutellarin groups. The cells was pretreated with Edaravone (100 μM), Scutellarin (0.54 mM), and Edaravone + Scutellarin 1 h at 37°C in a humidified incubator under 5% CO_2_. The dosage of Edaravone and Scutellarin used was based on cell viability assay when the drugs were used separately (data not provided). After incubation, the medium was discarded and the cells were washed with PBS, and then incubated with LPS (1 μg/ml, Sigma-Aldrich, MO, USA) for 3 h. The culture medium was replaced with basic DMEM before treatment. For controls, the medium was replaced with basic DMEM incubated in a chamber 95% air 5% CO_2_. Finally, proteins were extracted for Western blot analysis.

### Double immunofluorescence labeling in the cerebrum and BV-2 cells

A total of 15 rats of various experimental groups were used for double immunofluorescence labeling: 1, 3, 7 days (n = 5 at each time point). Following deep anesthesia with 6% sodium pentobarbital, the rats were sacrificed by perfusion with 2% paraformaldehyde in 0.1 M phosphate buffer. The brain was removed and paraffin embedded. Coronal sections of 7 μm thickness were cut on a microtome (Model: 2165; Leica, Bensheim, Germany).The sections were rinsed with phosphate-buffered saline (PBS). For blocking of non-specific binding proteins, tissue sections were incubated in 5% normal goat serum diluted in PBS for 1 h at room temperature (22–24°C). After discarding the serum, the sections were incubated in a humidified chamber with primary polyclonal antibody iNOS (mouse monoclonal IgG 1:100) (BD Pharmingen, San Jose, CA USA; Cat. No. 610432), TNF-α (rabbit polyclonal IgG 1:100) (Chemicon International, Temecula, CA, USA; Cat. No. AB1837P), and IL-1β (rabbit polyclonal IgG 1:100) (Chemicon International; Cat. No. AB1832P) diluted with PBS overnight at 4°C. Following washing in PBS, sections were incubated, respectively, with fluorescent secondary antibodies: Cy3-conjugated secondary antibody and FITC-conjugated lectin (*Lycopersicon esculentum*) that labels both microglia and blood vessel endothelial cells for 1 h at room temperature. After 3 rinses with PBS, the sections were mounted with a fluorescent mounting medium containing 4’,6-diamidino-2-phenylindole (DAPI) (Sigma, USA; Cat. No. F6057). Colocalization was observed by confocal microscopy (Fluoview 1000, Olympus Company Pte. Ltd., Tokyo, Japan). Details of antibodies used are given in Table 2.

**Table 2:** **Antibodies used for Western blotting and staining**

**Antibody**	**Host**	**Source**	**Catalog number /RRID**
iNOS	Mouse monoclonal	BD Pharmingen San Jose, CA USA	610432, RRID:AB_397808
TNF-α	Rabbit polyclonal	Chemicon, Temecula, CA, USA	AB1837P, RRID: AB_2204499
IL-1β	Rabbit polyclonal	Chemicon, Temecula, CA, USA	AB1832P, RRID:AB_2124750
β-actin	Mouse monoclonal	Sigma-Aldrich, MO, USA	A5441, RRID:AB_476744

BV-2 cells were fixed with 4% paraformaldehyde in 0.1 M PBS for 20 min. Following rinsing with PBS, the coverslips with adherent cells were used for immunofluorescence staining. In each group, BV-2 cells were incubated with the primary antibodies as described above overnight 4°C. Subsequently, the cells were incubated in FITC/Cy3-conjugated secondary antibodies for 1 h at room temperature. After washing, the coverslips were mounted using a fluorescent mounting medium with DAPI. All images were captured using a confocal microscope. The isotopic control confirmed the specificity of all primary antibodies used (data not shown).

### Western blotting analysis for MCAO tissues and BV-2 cells

A total of 105 rats were used for Western blotting analysis. The sham-operated and MCAO rats given saline, Edaravone and/or Scutellarin injections were sacrificed at 1 day (n = 5), 3 days (n = 5) and 7 days (n = 5), respectively. The control or ischemic cortex derived from each group was frozen in liquid nitrogen and stored at −80°C. Tissue samples from various groups were homogenized with protein extraction reagent (Pierce, IL, USA) containing protease inhibitors. For BV-2 cells of each group, the cells were lysed with lysis buffer, mechanically scraped off with a rubber scraper and centrifuged at 13,000 rpm for 25 min. Protein concentrations of both tissues and BV-2 cells were determined by using a protein assay kit (Bio-Rad, Hercules, CA, USA; Cat. No. 500–0002). Samples of supernatants containing 50 μg protein of tissues or 40 μg protein of BV-2 cells were loaded and heated to 95°C for 5 min, and were separated by sodium dodecyl sulfate-poly-acrylamide gel electrophoresis in 10% or 12% gels, in a Mini-Protein II apparatus (Bio-Rad, CA, USA). Protein bands were electroblotted onto polyvinylindene difluoride (PVDF) membrane and blocked with non-fat dried milk for 1 h. The membranes were incubated with iNOS (mouse monoclonal IgG 1:1000) (BD Pharmingen, San Jose, CA, USA; Cat. No. 610432), TNF-α (rabbit polyclonal IgG 1:1000) (Chemicon, International, Temecula, CA, USA; Cat. No. AB1837P), IL-1β (rabbit polyclonal IgG 1:1000) (Chemicon International; Cat. No. AB1832P), and β-actin (mouse monoclonal IgG 1:10000) (Sigma; Cat. No. A5441) primary antibodies diluted in Tris-Buffered Saline-0.1% Tween (TBST) overnight at 4°C. They were then incubated with the secondary antibodies, either with horseradish peroxidase (HRP) conjugated anti-rabbit IgG (dilution 1:3000) (Thermo Scientific; Cat. No. 31460) or anti-mouse IgG (dilution 1:20000) (Thermo Scientific; Cat. No.31430). Protein was detected by chemiluminescence kit (GE Healthcare UK Limited, Bucks, UK) following the manufacturer’s instructions and developed on the film. The band intensity was quantified in Image J software (National Institutes of Health, NIH, USA). All experiments were repeated at least in triplicate.

### Measurement of reactive oxygen species by flow cytometry

Intracelluar ROS production in BV-2 cells of different groups was evaluated by detecting the fluorescence intensity of 20, 70-dichlorofluorescene, the oxidized product of the fluoroprobe 5-(and 6)-chloromethyl-20, 70-dichlorodihydrofluorescein diacetate (CM-H2DCFDA, Molecular Probes, Invitrogen; Cat. No. C6827) following the manufacturer’s instruction. The amount of ROS production was considered to be directly proportional to fluorescence intensity given as cell counts and fluorescence intensity at the y-axis in the flow cytometry.

### Real time measurement of free nitric oxide

BV-2 cells were treated as described above and the cells were seeded directly onto glass chamber. NO production was measured by NO detection Kit for fluorescence microscopy (Enzo Life Science, NY, USA; Cat. No. ENZ-51013-200) according to the manufacturer’s instruction.

### Statistical analyses

The data are presented as mean ± standard deviation (±SD). Statistical significance was evaluated by one-way analysis of variance (ANOVA) followed by post-hoc test. The difference was considered statistically significant when *p* <0.05. SPSS 16.0 statistical software was used to analyze data.

## Additional files

Additional file 1: Figure S1. Treatment of MCAO rats with drugs E, SH and E + SH resulted in the reduction of iNOS expression in activated microglia. Confocal images showing the expression of iNOS (red) in lectin^+^ microglia (green) in the penumbral zones of MCAO rat brain (D-F) and following treatment with E (G-I), SH (J-L) and E + SH (M-O) (n = 5 for each group). Increase in the expression of iNOS (E) can be observed in the activated microglia (D) in MCAO rat brain. A marked reduction of iNOS expression (H, K, N) was observed in activated microglia (G, J, M) 7 days following treatment of MCAO rats with drugs E or SH and in combination. DAPI – blue. Scale bars in A-O: 50 μm. Additional file 2: Figure S2. Treatment of MCAO rats with drugs E, SH and E + SH resulted in the reduction of TNF-α expression in activated microglia. Confocal images showing the expression of TNF-α (red) in lectin^+^ microglia (green) in the penumbral zones of MCAO rat brain (D-F) and in the penumbral zones of MCAO rat brain following treatment with E (G-I), SH (J-L) and E + SH (M-O) (n = 5 for each group). An obvious increase in the expression of TNF-α (E) can be observed in the activated microglia (D) in MCAO rat brain. A marked reduction of TNF-α expression (H, K, N) was observed in activated microglia (G, J, M) 7 days following treatment of MCAO rats with drugs E or SH and in combination. DAPI – blue. Scale bars in A-O: 50 μm. Additional file 3: Figure S3. Effects of Edaravone and Scutellarin on viability of BV-2 cells. Showing cell viability of BV-2 cells treated with E and S for 1 hour. A combined concentration of Edaravone (in the range of 50 μM to 200 μM) and Scutellarin (in the range of 0.27 mM to 1.1 mM) did not result in any significant cell death in comparison to no drug controls. For further *in vitro* studies, an Edaravone concentration of 100 μM and Scutellarin concentration of 0.54 mM was used.
