# Supplementary figures and images for: Anti-inflammatory effects of Edaravone and Scutellarin in activated microglia in experimentally induced ischemia injury in rats and in BV-2 microglia
Source: BMC Neurosci. 2014 Nov 22;15:125. doi: 10.1186/s12868-014-0125-3 (PMC4247200; doi:10.1186/s12868-014-0125-3)

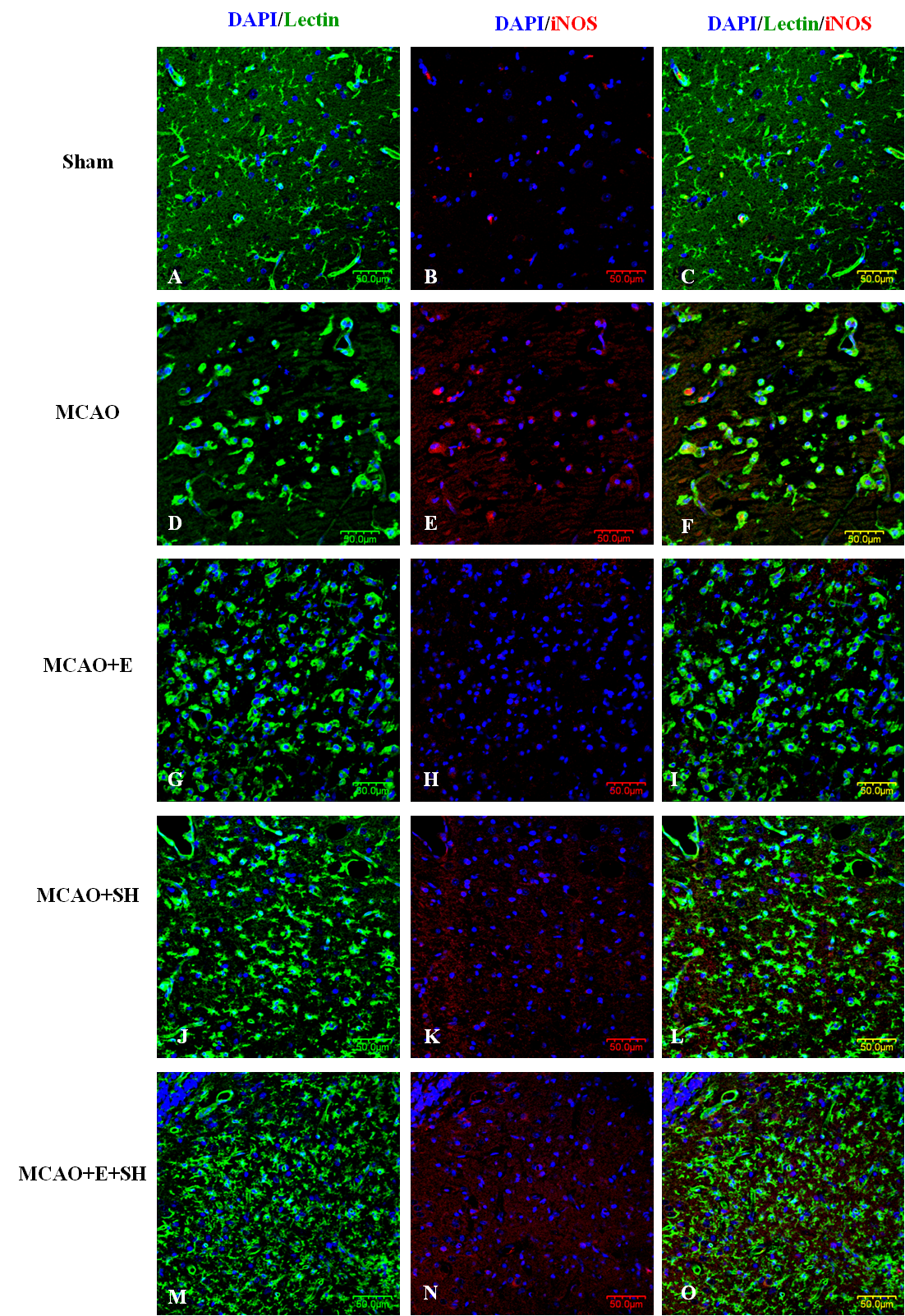

Supplement: Additional file 1: Figure S1. — Treatment of MCAO rats with drugs E, SH and E + SH resulted in the reduction of iNOS expression in activated microglia. Confocal images showing the expression of iNOS (red) in lectin+ microglia (green) in the penumbral zones of MCAO rat brain (D-F) and following treatment with E (G-I), SH (J-L) and E + SH (M-O) (n = 5 for each group). Increase in the expression of iNOS (E) can be observed in the activated microglia (D) in MCAO rat brain. A marked reduction of iNOS expression (H, K, N) was observed in activated microglia (G, J, M) 7 days following treatment of MCAO rats with drugs E or SH and in combination. DAPI – blue. Scale bars in A-O: 50 μm. [file 12868_2014_125_MOESM1_ESM.tiff]

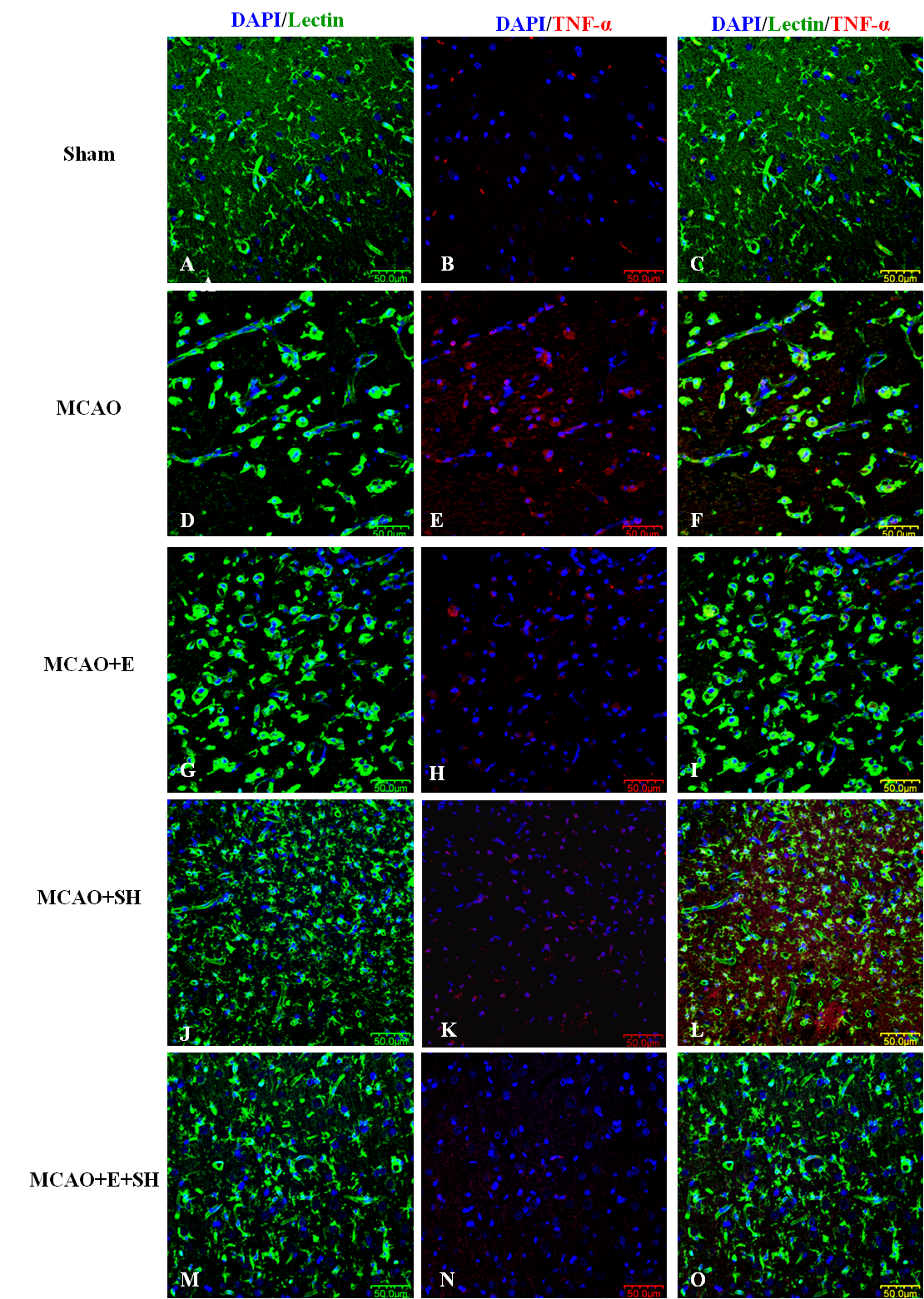

Supplement: Additional file 2: Figure S2. — Treatment of MCAO rats with drugs E, SH and E + SH resulted in the reduction of TNF-α expression in activated microglia. Confocal images showing the expression of TNF-α (red) in lectin+ microglia (green) in the penumbral zones of MCAO rat brain (D-F) and in the penumbral zones of MCAO rat brain following treatment with E (G-I), SH (J-L) and E + SH (M-O) (n = 5 for each group). An obvious increase in the expression of TNF-α (E) can be observed in the activated microglia (D) in MCAO rat brain. A marked reduction of TNF-α expression (H, K, N) was observed in activated microglia (G, J, M) 7 days following treatment of MCAO rats with drugs E or SH and in combination. DAPI – blue. Scale bars in A-O: 50 μm. [file 12868_2014_125_MOESM2_ESM.tiff]

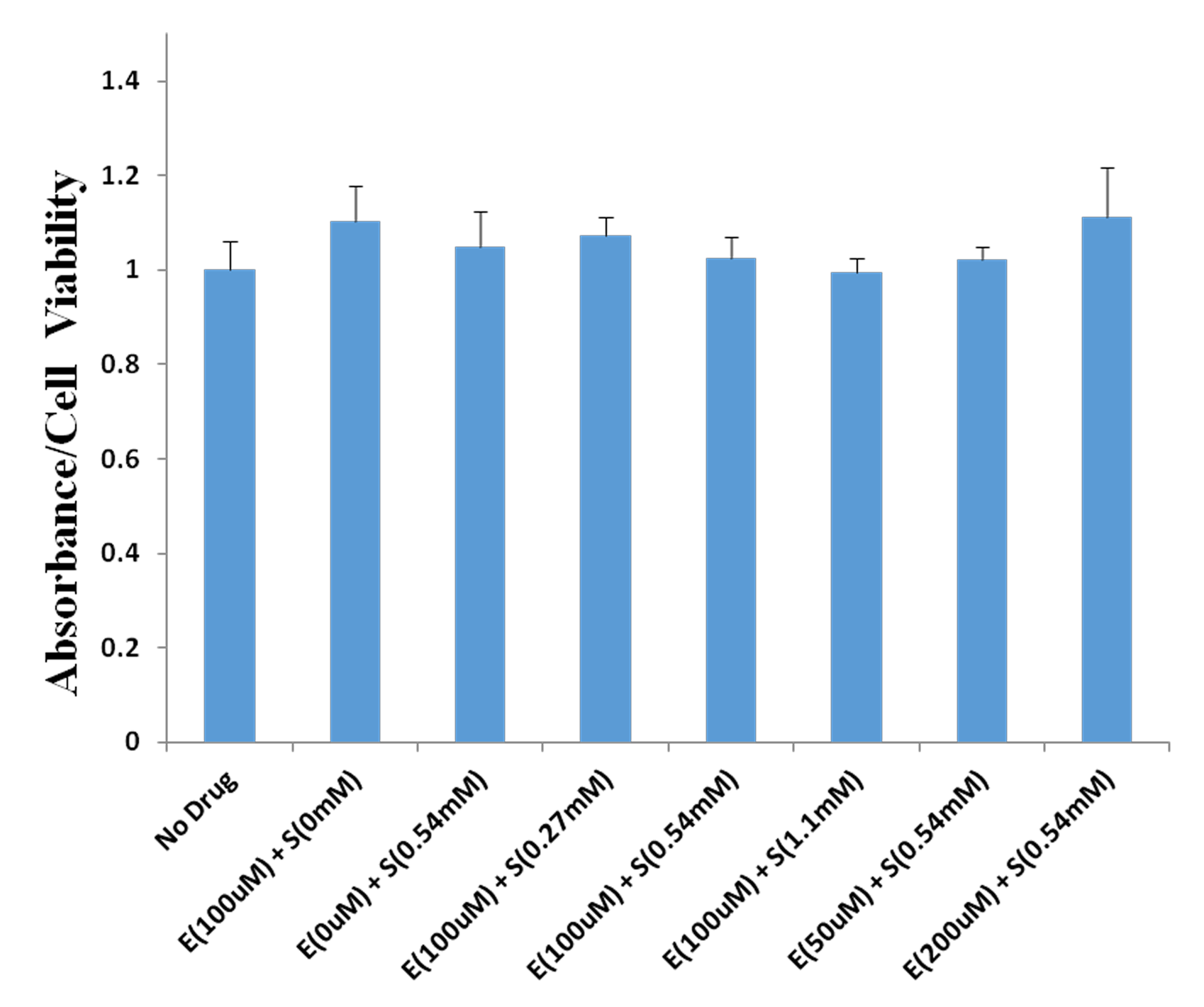

Supplement: Additional file 3: Figure S3. — Effects of Edaravone and Scutellarin on viability of BV-2 cells. Showing cell viability of BV-2 cells treated with E and S for 1 hour. A combined concentration of Edaravone (in the range of 50 μM to 200 μM) and Scutellarin (in the range of 0.27 mM to 1.1 mM) did not result in any significant cell death in comparison to no drug controls. For further in vitro studies, an Edaravone concentration of 100 μM and Scutellarin concentration of 0.54 mM was used. [file 12868_2014_125_MOESM3_ESM.tiff]
